# Protocol for “Seal or Varnish?” (SoV) trial: a randomised controlled trial to measure the relative cost and effectiveness of pit and fissure sealants and fluoride varnish in preventing dental decay

**DOI:** 10.1186/1472-6831-12-51

**Published:** 2012-11-20

**Authors:** Ivor Gordon Chestnutt, Barbara Lesley Chadwick, Simon Hutchings, Rebecca Playle, Timothy Pickles, Catherine Lisles, Nigel Kirkby, Maria Zeta Morgan, Lindsay Hunter, Ceri Hodell, Beverely Withers, Simon Murphy, Sarah Morgan-Trimmer, Deborah Fitzsimmons, Ceri Phillips, Jacqueline Nuttall, Kerenza Hood

**Affiliations:** 1 Applied Clinical Research and Public Health, Cardiff University School of Dentistry, Heath Park, Cardiff, CF14 4XY, UK; 2 South East Wales Trials Unit, School of Medicine, Cardiff University, Neuadd Meirionnydd, Heath Park, Cardiff, CF14 4YS, UK; 3 Community Dental Service, Cardiff and Vale University Health Board, Whitchurch Hospital, Cardiff, CF14 7XB, UK; 4 DECIPHer, School of Social Sciences, Cardiff University, 1–3 Museum Place, Cardiff, CF10 3BD, UK; 5 Swansea Centre for Health Economics, College of Human and Health Sciences, Swansea University, Swansea University, Singleton Park, Swansea, SA2 8PP, UK

**Keywords:** Dental caries, Clinical trial, Pit and fissure sealants, Fluoride varnish, Preventive dentistry, Oral health

## Abstract

**Background:**

Dental caries remains a significant public health problem, prevalence being linked to social and economic deprivation. Occlusal surfaces of first permanent molars are the most susceptible site in the developing permanent dentition. Cochrane reviews have shown pit and fissure sealants (PFS) and fluoride varnish (FV) to be effective over no intervention in preventing caries. However, the comparative cost and effectiveness of these treatments is uncertain. The primary aim of the trial described in this protocol is to compare the clinical effectiveness of PFS and FV in preventing dental caries in first permanent molars in 6-7 year-olds. Secondary aims include: establishing the costs and the relative cost-effectiveness of PFS and FV delivered in a community/school setting; examining the impact of PFS and FV on children and their parents/carers in terms of quality of life/treatment acceptability measures; and examining the implementation of treatment in a community setting.

**Methods/design:**

The trial design comprises a randomised, assessor-blinded, two-arm, parallel group trial in 6–7 year old schoolchildren. Clinical procedures and assessments will be performed at 66 primary schools, in deprived areas in South Wales. Treatments will be delivered via a mobile dental clinic. In total, 920 children will be recruited (460 per trial arm). At baseline and annually for 36 months dental caries will be recorded using the International Caries Detection and Assessment System (ICDAS) by trained and calibrated dentists. PFS and FV will be applied by trained dental hygienists. The FV will be applied at baseline, 6, 12, 18, 24 and 30 months. The PFS will be applied at baseline and re-examined at 6, 12, 18, 24, and 30 months, and will be re-applied if the existing sealant has become detached/is insufficient. The economic analysis will estimate the costs of providing the PFS versus FV. The process evaluation will assess implementation and acceptability through acceptability scales, a schools questionnaire and interviews with children, parents, dentists, dental nurses and school staff. The primary outcome measure will be the proportion of children developing new caries on any one of up to four treated first permanent molars.

**Discussion:**

The objectives of this study have been identified by the National Institute for Health Research as one of importance to the National Health Service in the UK. The results of this trial will provide guidance on which of these technologies should be adopted for the prevention of dental decay in the most susceptible tooth-surface in the most at risk children.

**Trial registrations:**

ISRCTN ref: ISRCTN17029222 EudraCT: 2010-023476-23 UKCRN ref: 9273

## Background

Despite the decline in the prevalence of dental decay in the United Kingdom in the last three decades, 57% of 15 year-olds still have dental caries (tooth decay) sufficiently severe to require a filling or extraction [1]. Dental caries is uneven in its distribution in the population and has been shown by numerous studies to be closely linked to social and economic deprivation [2], with a three-fold difference in disease burden from most to least deprived [3]. Within the mouth, the majority of detected incremental decay is to be found on the pit and fissure surfaces of molar teeth in children [4] and adults [5]. These facts dictate the need for caries-prevention technology, targeted at the most susceptible tooth surfaces in the most susceptible members of the population. Two competing technologies have the potential to fulfill this role, pit and fissure sealants and fluoride varnishes.

Pit and fissure sealants comprise a Bis-GMA resin, which is applied to the occlusal surface of the tooth using acid-etch technology. They work by physically obliterating the pit and fissure system which harbours cariogenic organisms and thereby inhibit the initiation of caries. First developed in the 1960s, they are an established technology and widely used in clinical practice. Numerous studies have investigated the clinical effectiveness of fissure sealants and this has been the subject of a Cochrane review. A meta-analysis of seven studies comparing sealed teeth to untreated controls demonstrated caries reductions ranging from 87% at 12 months to 60% at 48–54 months [6].

Fluoride varnishes have also been marketed since the 1960s and comprise a topical medication which is painted onto the tooth surface. They contain a high concentration of fluoride (22,600 ppm) and are licensed for application by dental professionals. The varnish forms a quick-setting base which subsequently releases fluoride. Fluoride acts to prevent caries by inhibiting the demineralisation and encouraging the remineralisation of dental enamel. A Cochrane review suggested a pooled prevented fraction estimate of 46% (95%CI 30%-63%) when fluoride varnish is tested against no treatment controls [7].

In relation to the cost-effectiveness of technologies in this field, the evidence base is generally sparse with relatively few attempts to assess the relative worth of techniques to prevent dental decay. Those that have been undertaken tend to suffer from design and methodological flaws. However, one US study [8] assessed the 4-year incremental cost-utility of sealing first permanent molars, compared with non-sealed molars, of 6-year-olds from a societal perspective and identified the group of teeth or children in whom sealants are most cost-effective. They concluded that sealants improved overall utility of first permanent molars after 4 years; that the cost-utility ratio of sealing the first permanent molar varied by arch and type of utilisers; and that sealing first permanent molars in lower dental utilisers was the most cost-effective approach for prioritizing limited resources. However, this study was a comparison between sealing and non-sealing, as opposed to a comparison between different preventive technologies. Thus, while the clinical effectiveness of the technologies (PFS and FV) as caries-preventive measures is established when compared to no treatment controls, a significant question remains unanswered: What is the relative clinical and cost-effectiveness of these technologies when compared to each other?

The application of PFS in particular is an involved procedure, the success of which is dependent on clinical delivery in a dental-chair. The acid-etch necessary for their long-term adherence to the tooth surface is critically dependant on maintaining a dry-field during application. The clinical technique involves the use of rotary dental instruments to clean the tooth surface, suction devices and a compliant patient. In contrast, FV application simply involves drying the tooth and painting with a brush/applicator. FV can be applied by less skilled dental personnel (nurses) whereas PFS requires the involvement of a dentist or dental hygienist. If FV were proven to be an acceptable alternative to PFS (in terms of clinical, cost and patient acceptability criteria) then the potential benefits in terms of health improvement and the effective and efficient delivery of services would be enormous.

Preventive dental methods can be very effective in reducing tooth decay in children. A recent Cochrane review has compared the effectiveness of pit and fissure sealants with fluoride varnishes in the prevention of dental decay on occlusal surfaces [9]. Hiiri and colleagues concluded that while there was some evidence of the superiority of PFS over FV in occlusal decay prevention it remains unclear to what extent there is a difference between the effectiveness of PFS and FV [9]. Importantly from the perspective of the National Health Service (NHS) in the United Kingdom, there is insufficient evidence on which to make recommendations for clinical practice and which (PFS or FV) represents the most effective technology. The review did not specifically address the relative efficiency of the technologies.

There is therefore good quality secondary research evidence which has identified the need for the study described in this protocol.

From a societal perspective, the cost of treating dental caries poses a substantial burden on dental services and individuals throughout life. Avoiding the pain and suffering associated with dental caries is desirable, as is avoiding the impact of dental disease on the quality of life of affected individuals. Dental restorations in permanent teeth in childhood require maintenance throughout life. The outcome of this trial has the potential to benefit both those participating and, by extrapolation, the one in three children in Great Britain who have experienced dental decay by eleven years of age.

The Community Dental Service (CDS) of Cardiff and Vale University Health Board provides preventive dental care to children attending primary schools in areas of social and economic deprivation under the Welsh Government’s “Designed to Smile” programme. Under this programme Mobile Dental Clinics (MDCs) visit schools at regular intervals. This programme provides the vehicle for delivery of this trial.

### Trial aim

The overall aim of the study is to identify and compare the relative clinical and cost-effectiveness of two established technologies, pit and fissure sealants (PFS) and fluoride varnish (FV) for the prevention of dental caries in first permanent molar teeth in children aged 6–7 years.

### Trial objectives

The objectives of the SoV trial are as follows:

#### Primary objective

To compare the clinical effectiveness of PFS and FV in preventing dental caries in first permanent molars in 6–7 year-olds, as determined by:

• The proportion of children developing new caries on any one of up to four treated first permanent molars

• The number of treated first permanent molar teeth caries-free at 36 months

#### Secondary objectives

• To establish the costs and budget impact of PFS and FV delivered in a community/school setting and the relative cost-effectiveness of these technologies

• To examine the impact of PFS and FV on children and their parents/carers in terms of quality of life/treatment acceptability measures.

• To examine the implementation of treatment in a community setting with respect to the experience of children, parents, schools and clinicians.

## Methods/design

### Study design

This is a randomised, assessor-blinded, two-arm, parallel group trial in 6–7 year old children. A total of 920 participants will be randomised to receive either PFS or FV. Clinical procedures and assessments will be performed at approximately 66 schools in South Wales via the use of a mobile dental clinic.

**Figure 1:**
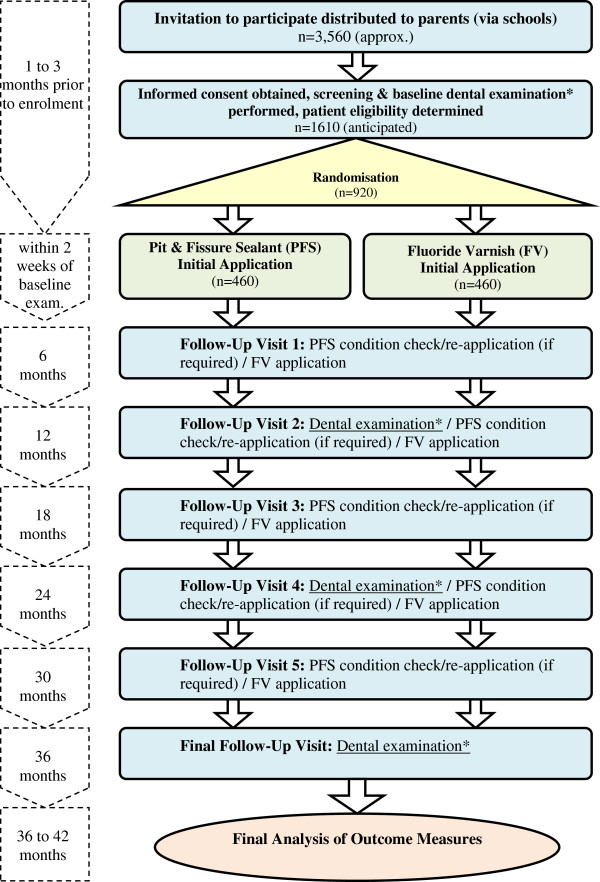
Trial Schema.

The trial schema is shown in Figure 1.

### Setting and study participants

The target population is children aged 6–7 years attending primary schools in areas of social and economic deprivation. All children in these schools are deemed at high caries-risk according to Scottish Intercollegiate Guidelines Network (SIGN) [10] /British Society of Paediatric Dentistry (BSPD) guidelines [11] and qualify for PFS/FV application. With parental consent and child assent, children aged 6–7 years who have at least one erupted non-carious first permanent molar tooth will be randomly allocated to receive either PFS or FV.

### Participant selection

Children will be considered eligible to join the trial if they meet all of the following inclusion criteria and none of the exclusion criteria. Certain inclusion and exclusion criteria (where identified) require evaluation by a dentist during a baseline clinical examination. Only children that are considered to meet all other inclusion/exclusion criteria will undergo a baseline clinical examination.

#### Inclusion criteria

• Children aged 6–7 years, attending the schools participating in the current Cardiff and Vale University Health Board “Designed to Smile” Programme

• Children for whom the person with parental responsibility has provided written informed consent

• Children with at least one fully-erupted caries-free first permanent molar (determined at baseline examination)

#### Exclusion criteria

• Children whose medical history precludes inclusion (i.e. those with a history of hospitalisation for asthma, or severe allergies, or allergy to Elastoplast; determined from Medical History Form completed by parents)

• Children with known sensitivity to colophony (kolophonium), or any of the product ingredients (e.g. methylacrylate in PFS; determined from Medical History Form completed by parents)

• Children with ulcerative gingivitis or stomatitis (determined at baseline examination)

• Children with any facial or oral infections e.g. cold sores (determined at baseline examination)

• Children with any abnormality of the lips, face or soft tissues of the mouth that would cause discomfort in the provision of PFS/FV (determined at baseline examination)

• Children who are showing obvious signs of systemic illness (e.g. colds, ‘flu’, chicken pox etc.) (determined at baseline examination)

• Children currently participating in another clinical trial involving an investigational medicinal product (IMP; determined from Medical History Form completed by parents).

### Trial interventions

The two technologies to be evaluated in this trial (PFS and FV) are well established and have been used routinely to prevent dental caries for several decades. Eligible participants will be randomised to receive either PFS or FV, and will remain on the intervention to which they have been randomised for the duration of the study.

#### Pit and fissure sealant (PFS)

The PFS used for evaluation in the study is Delton® Light Curing Opaque Pit & Fissure Sealant (Dentsply Ltd; CE0086). We chose this product as a commonly used PFS and that used in the existing community dental programme. PFS will be supplied as 2.7 ml bottles for multiple applications and applied topically as a thin layer to the occlusal surface of eligible teeth.

Initial application of PFS will occur within 2 weeks of the baseline dental examination, and will be performed by a suitably qualified and trained dental hygienist according to the conventional clinical protocol established by the CDS. The condition of the PFS will be re-examined at 6, 12, 18, 24, and 30 months, and will be re-applied if the existing sealant has become detached, or if attachment is considered insufficient. Newly erupting FPMs will have PFS applied in the course of the trial.

All applications of PFS will be documented on a treatment record form, which will capture the date, batch number, patient ID number and number of sealants (i.e. teeth) applied.

#### Fluoride varnish (FV)

The FV used for evaluation in the study is Duraphat® 50 mg/ml dental suspension (Colgate-Palmolive (UK) Ltd; PL 00049/0042), equivalent to 22,600ppm fluoride. We chose this product as a commonly used FV and that used in the existing community dental programme. FV will be supplied as 10 ml tubes for multiple applications and applied topically as a thin layer to the pits, fissures and smooth surfaces of eligible teeth. As per the Duraphat Summary of Product Characteristics (SmPC), dosage per single application will not exceed 0.4 ml.

Initial application of FV will occur within 2 weeks of the baseline dental examination, and will be performed by a suitably qualified and trained dental hygienist according to the conventional clinical protocol established by the CDS. FV will be re-applied at 6, 12, 18, 24, and 30 months.

All applications of FV will be documented on a treatment record form, which will capture the date, batch number, patient ID number and number of applications (i.e. teeth) performed.

Where children enrolled onto the study are registered with a General Dental Practitioner (GDP), the GDP will be informed of the child’s participation in the study and requested to not apply either PFS/FV (or other fluoride-based treatment) for the duration of the trial.

### Outcome measures and follow-up of study participants

**Figure 2:**
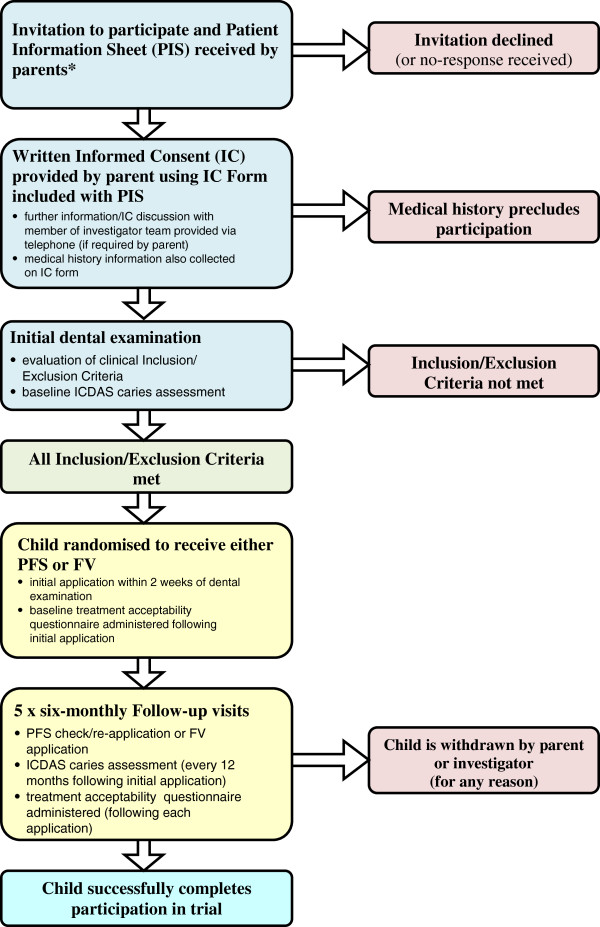
Participant flow through the trial.

Participant flow through the trial is shown in Figure 2.

### Clinical evaluation

Participants will undergo a clinical examination using standard visual caries diagnosis (both enamel and dentinal level) at baseline and 12, 24 and 36 months by a trained and calibrated dentist, blind to treatment allocation. All fully-erupted non-carious first permanent molars (FPM) will be treated.

#### ICDAS caries assessment

Caries status will be assessed at baseline for all children considered eligible. Caries status will be assessed and recorded by trained and calibrated dentists using conventional diagnostic caries criteria at the d1/D1- d3/D3 level in accordance with nationally recognised diagnostic criteria [12]. Data will be recorded using charts specifically designed for collection of ICDAS dental codes [13].

In addition to baseline, a clinical examination including ICDAS caries assessment will be performed at 12 month intervals for 36 months.

As part of the annual caries assessment approximately 5% of study participants will be re-examined to determine intra-examiner reproducibility.

#### Medical history and caries risk related habits

The medical history of the child will be ascertained by asking the parents to complete a medical history form, which will collect data specifically relating to: allergies; asthma (including whether this has resulted in hospitalisation); sensitivity to constituents of either PFS or FV; if the child is currently participating in a clinical trial involving an IMP. Whether or not the child is registered with a General Dental Practitioner will also be ascertained.

Following enrolment onto the trial, any changes to the child’s relevant medical history (as described above) will be identified by enclosing a brief Medical History Follow-Up Form with the postal questionnaires sent to parents on an annual basis during the trial.

For children deemed eligible to participate in the trial, information relating to caries risk related habits will be obtained via a postal questionnaire sent to parents at baseline, 12, 24 and 36 months post treatment.

### Health economics assessment

The economic analysis will estimate the costs of providing the PFS versus FV, and the consequences of the scheme for the NHS, children and their families, education and society.

In principle the following analyses will be undertaken:

• Costs for each trial participant will be calculated. Number and frequency of service utilisation will be multiplied by the relevant unit cost (derived from published sources: [14,15] to produce a total cost per participant

• Travel costs and other costs to families associated with provision of PFS and FV will be collected using structured questions

• Assessment of the total cost of PFS and FV including potential costs of treatments avoided.

The identification and collection of costs will be undertaken using the following methods.

#### National health service (NHS)

Data on the use of community health dental resources will be collected through structured interviews with key relevant dental and finance staff and the main trial team. A micro-costing exercise will also be undertaken to assess direct costs to the NHS. This will include staff resources (e.g. number, grades of staff), treatment/appointment duration; equipment and materials used. These will be logged on resource utilisation recording sheets at each clinic and costed using published unit costs [14,15] and list prices e.g. British National Formulary [16].

#### Children and families

The costs to families (including the child) will be collected using a Parental Resource Utilisation (PRU) questionnaire, which will be combined with questions relating to Caries Risk Related Habits to form a single postal ‘Dental Health’ questionnaire to be completed by parents. This brief questionnaire will capture information on any time away from work/other paid activities to attend the child’s appointment or other dental appointments; any medication for dental-related problems and normal dental hygiene practice at home for the child. In addition, parental occupation will be captured. As with Caries Risk Related Habits, this information will be collected at baseline, 12, 24 and 36 months post treatment.

The health-related quality of life (HRQoL) of children will be measured using the CHU-9D questionnaire [17,18]. The CHU-9D questionnaire will be sent to parents with instructions to ask the child to complete it, providing assistance if required. CHU-9D questionnaires will be sent out at 12, 24 and 36 months following the initial treatment. Utility values will be calculated to derive quality adjusted life years (QALYs).

#### Education

Data on the use of school resources will be collected through the administration of a structured questionnaire with the headteacher from participating schools. This will include time of the child away from classroom/usual school activities, school administration time, teacher time, other school personnel time and other school resources utilised. This information will be collected alongside the collection of information via the headmaster questionnaire survey on the acceptability, feasibility and sustainability of the programme in Schools.

#### Implementation costs

Costs of implementing the interventions are not applicable to this study as the MDC is already established. However, the analysis will take into account the depreciation costs of the clinic over the trial period and beyond as part of the sensitivity analysis.

#### Research costs

These will be separated from the other costs incurred to provide clarity. The research costs incurred as a result of establishing the study, training, running the evaluation, completing questionnaires will be collected through discussion with the main trial team.

### Process evaluation

The process evaluation for the study will address two secondary outcomes: treatment acceptability and implementation in a community setting. Trial implementation will also be examined.

Treatment acceptability will be assessed in three ways.

During the clinical placement of the technologies under investigation, the indicators of patient acceptability/adverse outcomes: vomiting, crying, gagging, excessive arm/leg movements and other signs of distress [19] will be recorded by both the dental hygienist and dental nurse using an observational scale. Treatment acceptability will also be assessed from the child’s perspective through a Delighted-Terrible Faces (D-T) scale, completed by all children in the MDC immediately following the initial application of PFS/FV, and at each follow up visit. This is a modified version of the Delighted-Terrible Faces (D-T) Scale [20,21]. This scale has a child-friendly format with a range of ‘delighted’ to ‘terrible’ faces arranged in a five-point Likert scale, with minimal text. These scales will be analysed statistically as part of the secondary outcomes analysis.

In addition, qualitative interviews will be conducted with a subsample of children and parents to collect data on the quality of the experience of receiving sealant or varnish treatment, and to understand factors affecting acceptability such as taste, length of treatment, treatment setting and prior family dental experience. Quantitative data will be triangulated with each other and also with parent and child interviews. Children and their parents from each trial arm will be interviewed in order to compare the experience of PFS and FV treatment, and interviewees will also be drawn from higher and lower deprivation areas in order to examine the impact of socio-economic status on participant perceptions of the dental treatment.

The process evaluation interviews will also explore the implementation of the Seal or Varnish programme in a community setting, addressing factors such as the utility of using a school setting to deliver preventative treatment and trial implementation covering factors affecting recruitment, retention and potential bias. These data will be used to illuminate trial outcomes.

#### Interviews with children

A sample of schools participating in the trial will be selected for the purpose of conducting interviews with children receiving treatment. Schools will be stratified by size and free school meal (FSM) entitlement. Larger schools will be selected to ensure an adequate number of children can be sampled for paired interviews. Schools will also be sampled from the upper and lower FSM quartiles in order to collect date on children from higher and lower deprivation areas. Within each school, children will be stratified by trial arm and also by level of treatment acceptability (above and below the mean) based on their D-T scale responses. In total, 48 children will be interviewed face-to-face in a school setting, 24 in each trial arm. This sampling will enable three domains (deprivation, measured by FSM; trial arm and acceptability level) to be analysed both within each domain (e.g. factors associated with high and low acceptability) but also an investigation into how the three domains intersect with each other. The staff member(s) responsible for trial aspects at each school will assist in inviting the sampled children to attend a face-to-face interview in pairs; this method should ensure children feel more confident and relaxed in interviews. Interviews will be conducted with children in a school setting up to two weeks after their first and last treatment to ensure children will have a reasonably accurate recall of their experience of treatment. Interviewers will be provided with information about the initial D-T scale results for each interviewee prior to the interviews to inform how questions are asked. The scale results will not be discussed during interviews, however, to ensure confidentiality since the interviews will be in pairs.

#### Interviews with parents

Parents of sampled children will be interviewed, with 24 parents interviewed in each trial arm. Parents will be interviewed by telephone four to six weeks after their child’s first and last treatments where possible. Where parents of sampled children cannot be contacted, additional parents of participating children will be sampled to ensure an overall total of 48 parents are interviewed.

#### Questionnaires/interviews with schools

All schools will be asked to complete a questionnaire at the beginning of the study regarding their experience with implementation of the trial. At the end of the study a number of schools will be purposively sampled based on responses to the questionnaires. The member of staff within each participating school who has the most involvement in the trial will be invited to take part in a telephone interview to investigate the impact of the trial on the school. The implications of this for the acceptability and feasibility of the trial in a community setting will be determined.

#### Interviews with the dental team

The dental team delivering the treatment in schools will be interviewed annually in order to assess how the trial is implemented, factors which promote or impede implementation, perceptions of patient acceptability, and experiences of working with schools.

#### Interviews with non-participants

Up to 20 withdrawing parents will be contacted by letter seeking consent for a telephone interview. Data from these interviews will be compared by trial arm in order to examine reasons for leaving the study and any possible bias this might introduce. A sample of up to 20 non-responding and up to 20 non-consenting parents will also be contacted for interview. Data from these interviews will be used to explore any potential bias introduced into the study and to assess contextual factors affecting recruitment into the programme.

### Randomisation

Randomisation of participants will be stratified by school and balanced for gender and baseline caries levels using minimisation in a 1:1 ratio for treatments. An additional random component will be added to the minimisation algorithm [22] such that it is not completely deterministic. Randomisation will be undertaken by SEWTU remote from the trial sites.

### Sample size considerations

Data from a cohort study among primary school children under the care of the Cardiff and Vale University Health Board CDS was used to derive the caries incidence in children (mean age 6.5yrs) with at least one erupted first permanent molar [23]. These data showed that 40% had caries in one or more of their first permanent molars by the age of 10. Based on recent Cochrane reviews it is estimated that FV would reduce the 3 year incidence from 40% to 30% in this population [7], whereas PFS would reduce it further to 20% [6]. For an individually randomised trial at a power of 80% with a significance level of 5%, at least 313 children per group are required for a comparison of 20% vs 30% at 3 year follow-up.

The following assumptions have been made to ensure the necessary recruitment and retention: 3560 parents will be invited to consent to their child’s participation in the trial, associated with an estimated 55% refusal rate. Experience from the existing programme shows that 2% of consented children will refuse to cooperate to allow PFS placement and an estimated 1% will be excluded on medical grounds. An anticipated 40% of consented children will lack an erupted FPM and therefore be ineligible for randomisation.

The above attrition is estimated to leave 920 participants to be randomised to the technologies being evaluated (460 per arm). An 8% (n=204) per annum loss to follow-up has been assumed based on the current programme and a previous cohort study [23]. Finally a 5% absence at the final examination has been assumed, leaving 680 children for analysis at the final examination.

### Research governance / ethical considerations

Given the extensive clinical experience and relatively low risk nature of the two interventions under evaluation, several aspects of the MHRA/DH/MRC guidance on risk-based approaches to the management of Clinical Trials of Investigational Medicinal Products (CTIMPS) [24] were employed in the design and implementation of this trial (specifically, with regards to IMP management and adverse event reporting). Furthermore a risk-based approach to the informed consent process was developed in collaboration with a parent's group from a representative participant population [25].

Full ethical approval for this study has been obtained from the Research Ethics Committee for Wales (Ref: 11/MRE09/06). The study will be conducted in compliance with the following:

• Declaration of Helsinki [26]

• ICH Harmonised Tripartite Guideline for Good Clinical Practice [27]

• The Medicines for Human Use (Clinical Trials) Regulations 2004 (Statutory Instrument 2004 No. 1031) as amended by the Medicines for Human Use (Clinical Trials) Amended Regulations 2006 (Statutory Instrument 2006 No. 1928 and No. 2984) and Amended Regulations 2008 (Statutory Instrument 2008 No. 941) [28].

• Research Governance Framework for Health and Social Care (Welsh Assembly Government 2nd Edition, September 2009 and Department of Health 2nd Edition, July 2005) [29].

Two external bodies; a Data Monitoring and a Trial Steering Committee, will monitor study progress.

### Adverse events

**Figure 3:**
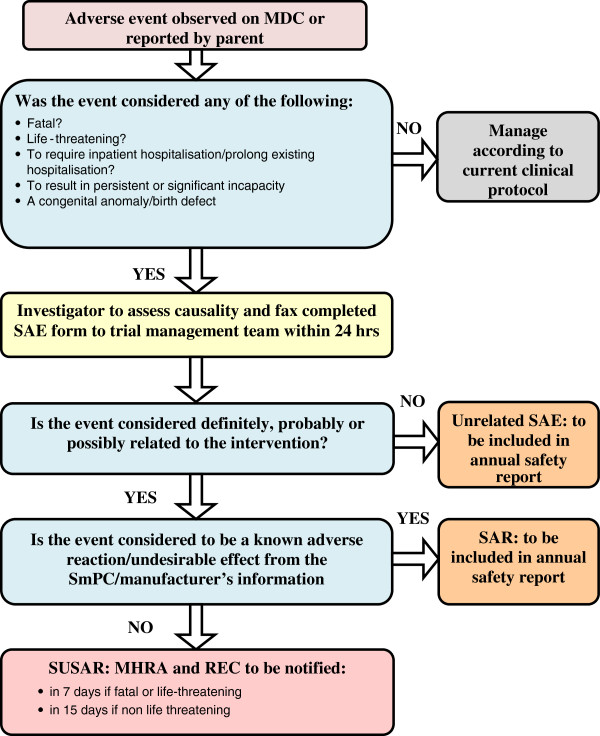
Flow chart for Adverse Event Reporting Procedures.

The procedure for reporting adverse events arising as a result of participation in the trial is show in Figure 3.

### Statistical analysis

The primary outcome measure will be the presence or absence of dental caries on any of the first permanent molars included in the trial at 36 months. Secondary outcomes include patient, hygienist and nurse rated treatment acceptability as well as sealant retention for those children in the sealant arm. Outcome measures for the health economic analysis include Health Related Quality of Life and Quality Adjusted Tooth Years.

### Primary outcome measurement

The presence or absence of dental caries and caries treatment on all surfaces of all teeth will be recorded using the ICDAS system [13]. Summed counts of surfaces and teeth with disease and or treatment are computed from these data. Counts for first permanent molars included in the trial will be calculated. These counts are then converted into a binary outcome at child level. The proportion of participants with any treated or untreated caries (at ICDAS level 4) in the first permanent molars included in the trial at the final follow-up examination will be compared between the two trial arms.

ICDAS equivalents to conventional DMFT (D3 caries into dentine level) will be calculated for the primary analysis; caries at earlier stages (level 2 and 3) will be used for secondary analysis of the primary outcome. Caries counts for the whole mouth will also be calculated for use in further exploratory analyses.

### Secondary outcome measurement

Patient rated treatment acceptability will be measured using the Delighted-Terrible (D-T) faces scale. This scale consists of five items rated 1–5 relating to the participants experience of treatment. The D-T scale score will be calculated as the sum of valid responses for individual items divided by the number of valid responses.

The hygienist and nurse rated treatment acceptability will be measured using the Adverse Outcomes (A-O) scale. This scale consists of 6 items scored 0–1. The A-O scale score will be calculated as the sum of valid responses for individual items divided by the number of valid responses.

The retention of sealants in the sealant arm will be derived from the treatment records. The proportion of sealants retained intact and partially intact will be reported at 1 year, 2 years and 3 year time points.

The presence and severity of hypomineralisation/hypoplasia in the first permanent molars will be recorded at each dental examination and the prevalence reported by treatment arm. Severely hypoplasic teeth will be excluded from the trial.

### Descriptive analysis

#### Analysis of primary outcome

The primary outcome for this study is the presence of filled or unfilled caries (at ICDAS level 4) on any 1 of up to 4 teeth included in the trial per child. As such the primary outcome is binary at child level. Logistic regression will be used to determine the difference between treatments. Covariates to be included in the primary model will include those variables used to balance the randomisation (gender and baseline caries) as well as the number of designated study teeth per child. Adjusted and unadjusted odds ratios from the logistic regression will be reported, the adjusted analysis will be taken as the primary outcome.

Since children for this study are to be recruited from schools there may be some possibility of school clustering effects. Schools are likely to be similar in terms of caries risk since they are all designated Community First schools, however multilevel modelling will be used to account for possible clustering at school level. ICC values will be reported.

The Complete Case (CC) population will be used for the primary outcome analysis.

#### Analysis of secondary outcomes

The distribution of the Patient Rated Treatment Acceptability (D-T) score and the Adverse Outcomes score will be examined for departures from normality. Rating scales typically exhibit positive skew however small departures from normality do not preclude parametric methods. Negatively skewed data will be transformed such that it conforms to a normal distribution.

Linear regression analysis will be used to model the difference between scores for the treatment groups. Covariates to be considered for inclusion in the model are gender, baseline caries, fluoride use, oral hygiene regimen and dietary sugar intake. Adjusted and unadjusted effect sizes and confidence intervals will be reported. The primary analysis for secondary outcome will be the adjusted analysis.

The data will be examined for possible school cluster effects and if significant, a 2-level linear regression analysis will be used to adjust for clustering. ICC values will be reported.

Appropriate summary measures for the scores will be reported in tables in original (untransformed) scale. Effect sizes and confidence intervals will be back transformed if required for ease of interpretation.

For those children in the sealant arm of the trial, sealant retention will be examined and reported. Secondary outcomes analysis will use the treatment groups as randomised using the CC population.

#### Secondary analysis of primary outcomes

An exploration of child and school level characteristics will be carried out using a 2-level logistic regression model. School level factors include size of school, postcode and participation in the oral health education programme. Child level factors include data collected from parent interviews on fluoride toothpaste use, oral hygiene routine and dietary sugar habits as well as Welsh Index of Multiple Deprivation [30].

Secondary analysis of the primary outcome will also be carried out as a per-protocol analysis on those participants who attend all treatment sessions (baseline and 5x6montly appointments where required) irrespective of randomisation assignment. Any dose response for number of fluoride treatment applications and sealant retention time on the primary outcome will also be investigated.

Multi-level regression analysis will also be carried out using tooth level data. A 2- or 3- level logistic regression model with tooth, child and possibly school will be used to examine caries incidence in the first permanent molars. Tooth levels factors such as upper or lower arch as well as child level factors will be included.

An investigation of the treatment differences on the earlier development of caries will also be examined using level 2 and 3 of the ICDAS classification of caries.

Data entry, management and analysis will be conducted centrally at the South East Wales Trials Unit (SEWTU).

### Time plan for the SoV trial

Participant recruitment began in July 2011 and is planned to continue to until December 2012. Children will be followed up for three years and therefore the last child will have their final follow-up assessment in December 2015.

## Discussion

The study will be conducted in primary schools in South Wales. Under the umbrella of the Welsh Oral Health Action Plan, the Community Dental Service (CDS) in Cardiff and Vale University Health Board delivers a primary school based PFS placement programme (‘Designed to Smile’) to approximately 3,300 6 year-olds annually. This programme, and its predecessor, have been in place since 2002. The programme involves Community First Schools, so designated by the Welsh Government on the basis of the high socioeconomic deprivation status of the school catchment area. The prevalence of dental caries in these areas is amongst the highest in the United Kingdom [31]. Permission to adapt the Designed to Smile programme for the purposes of this trial has been granted by the Chief Dental Officer for Wales.

A schools-based programme, utilising mobile dental clinics, is considered to have several advantages over conducting the proposed research in general dental practices.

The Designed to Smile programme is established and gives access to a cohort of high-risk children, experiencing the necessary burden of disease to allow an appropriate test of the technologies. This setting accesses children who might not otherwise routinely attend a general dental practice - attendance being linked to socioeconomic status [32]. In addition to having the necessary throughput of patients to achieve the required recruitment rate of at-risk children (as defined by SIGN / BSPD guidelines [10,11]), a schools-based setting will facilitate control over retention and follow-up. Furthermore, and perhaps most importantly, the CDS has established a relationship with the schools, parents and the children over many years which will obviously facilitate meaningful user and participant involvement in the study. In addition to service user representatives, staff involved in the existing CDS programme were consulted during the design and work-up of this trial, ensuring that it will be implemented and conducted in a way that is empathetic to the needs of the participating children, their parents and their schools.

On the basis that we are testing the relative clinical and cost-effectiveness of two established technologies, both of which have been proven clinically effective against placebo treatments [6,7], this trial will not contain a placebo control arm.

The objectives of this study have been identified by the National Institute for Health Research as one of importance to the National Health Service in the UK.

## Competing interests

The authors declare that they have no competing interests.

## Authors’ contributions

IGC, BLC, KH, SH and JN were responsible for drafting the protocol and are responsible for overall design and management the trial. RP, TP, NK and CL contribute to the statistical and data management. CH, BW, LH and MZM are responsible for clinical delivery of the study and provided details of the operalisation of the trial. SM and SM-T contribute to the qualitative and process evaluation elements and DF and CP are accountable for the health economic components of the work. All authors read and approved the final manuscript.

## Pre-publication history

The pre-publication history for this paper can be accessed here:

http://www.biomedcentral.com/1472-6831/12/51/prepub
